# The Unified Protocol for Transdiagnostic Treatment of Emotional Disorders Among Japanese Children: A Pilot Study

**DOI:** 10.3389/fpsyg.2021.731819

**Published:** 2021-11-25

**Authors:** Hiroko Fujisato, Noriko Kato, Hikari Namatame, Masaya Ito, Masahide Usami, Tomoko Nomura, Shuzo Ninomiya, Masaru Horikoshi

**Affiliations:** ^1^National Center for Cognitive Behavior Therapy and Research, National Center of Neurology and Psychiatry, Tokyo, Japan; ^2^Faculty of Human Sciences, University of Tsukuba, Ibaraki, Japan; ^3^Kohnodai Hospital, National Center for Global Health and Medicine, Chiba, Japan; ^4^Graduate School of Humanities and Sciences, Ochanomizu University, Tokyo, Japan

**Keywords:** child, transdiagnostic, Unified Protocol, anxiety, depression, cultural adaptation, cognitive behavioral therapy

## Abstract

At present, there is no established cognitive behavioral therapy (CBT) for treating emotional disorders in Japanese children. Therefore, we introduced the Unified Protocol for Transdiagnostic Treatment of Emotional Disorders in Children (UP-C) in Japan and adapted it to the Japanese context. We then examined its feasibility and preliminary efficacy using a single-arm pretest, posttest, follow-up design. Seventeen Japanese children aged between 8 and 12 years (female *n* = 11; male *n* = 6; *M* = 10.06 ± 0.97 years) with a principal diagnosis of anxiety, obsessive-compulsive, or depressive disorders, and their parents were enrolled in the study. The primary outcome was the overall severity of emotional disorders as assessed by psychiatrists using the Clinical Global Impression-Severity Scale. Secondary outcomes included child- and parent-reported anxiety symptoms, depressive symptoms, and functional status. No severe adverse events were observed. The feasibility was confirmed by the low dropout proportion (11.76%), high attendance proportion (children: 95.6%; parents: 94.6%), and sufficient participant satisfaction. Linear mixed models (LMMs) showed that the overall severity of emotional disorders and child- and parent-reported anxiety symptoms improved from pre-treatment to post-treatment, and that these treatment effects were maintained during the 3-month follow-up period. Additionally, child- and parent-reported functional status improved from pre-treatment to the 3-month follow-up. In contrast, child-reported depressive symptoms improved from pre-treatment to follow-up, but there was no significant change in parent-reported depressive symptoms between pre-treatment and other time points. These findings demonstrate the feasibility and preliminary efficacy of the Japanese version of the UP-C, suggesting that future randomized controlled trials (RCTs) are warranted (Clinical trial registration: UMIN000026911).

## Introduction

Emotional disorders, such as anxiety, depressive, and obsessive-compulsive disorders in children are by no means rare. Large epidemiological studies in Europe and the United States have shown that among children under the age of 13, the prevalence of anxiety, depressive, and obsessive-compulsive disorder is 6.6%, 2.7% (Bittner et al., 2007), and 1.8% (Canals et al., 2012), respectively. Epidemiological studies in Japan are limited, with only one study showing that 2.9% of children suffer from any type of depressive disorders (Denda, 2008). However, a meta-analysis of 41 studies conducted in 27 countries worldwide found that variability in prevalence estimates was not explained by the geographic location of the studies, suggesting that mental disorders affect a significant number of children and adolescents globally (Polanczyk et al., 2015).

Previous studies have found that childhood emotional disorders are a risk factor for school-related and interpersonal problems. For example, Ezpeleta et al. (2001) showed that children with anxiety or depressive disorders had more parent disabilities (i.e., disabilities related to interaction with parents and problems with chores), peer disabilities (i.e., disabilities in sibling or peer relationships), and educational disabilities (i.e., disabilities related to interaction with teachers, homework problems, disability in school performance, and suspension/expulsion) than children without any mental disorders. Canals et al. (2012) found that children with obsessive-compulsive disorder showed significant global functional impairment and lower academic performance compared to children without this disorder. Additionally, many studies have clarified that emotional disorders (symptoms) in childhood are sometimes maintained in the same form, and sometimes develop into other disorders (symptoms) during adolescence or adulthood (Aronen and Soininen, 2000; Bittner et al., 2007; Fullana et al., 2009; Cohen et al., 2018). For example, Cohen et al. (2018) showed that childhood anxiety predicted adolescent anxiety and depression, while childhood depression predicted adolescent depression. Bittner et al. (2007) clarified that childhood separation anxiety disorder predicted adolescent separation anxiety disorder, whereas childhood social phobia was associated with adolescent overanxious disorder, social phobia, and attention-deficit/hyperactivity disorder. Thus, emotional disorders in early childhood should not be overlooked as a temporary condition during the growth process. Early and appropriate treatment should be provided for the lifelong well-being and mental health of the children.

For emotional disorders, many disorder-specific cognitive behavioral therapy (CBT) programs have been developed and shown to be effective (Crowe and McKay, 2017). Therefore, CBT is recommended as a first-line non-pharmacological treatment for childhood emotional disorders (Higa-McMillan et al., 2016; Weersing et al., 2017; Freeman et al., 2018). For children, pharmacotherapy may not be suitable and may not regulate symptoms of emotional disorders, because many medications approved for adults have not been proven to work on children; additionally, some antidepressants often used for childhood emotional disorders may induce activation syndrome, especially in younger children (Luft et al., 2018). Therefore, the need for CBT is imperative. However, the concurrent and sequential comorbidity between anxiety and depression is common in children and adolescents (Garber and Weersing, 2010). The focus on disorder-specific CBT contrasts with high comorbidity between disorders. To address these practical problems, transdiagnostic CBT, treatments that address multiple disorders or problem sets using a single protocol, has been developed and the research on this approach has been accumulated. Potential strengths of transdiagnostic approaches include increased efficiency of training in and dissemination of evidence-based practices, reduced training and supervision costs for organizations and practitioners, improved fit to the way clinicians function in everyday practice, improved fit to the characteristics of referred youths and their treatment, and increased clinician and client satisfaction (Marchette and Weisz, 2017). Some meta-analyses showed that transdiagnostic CBT for adult populations are effective in reducing anxiety and depression with large effect sizes (Newby et al., 2015; García-Escalera et al., 2016). Although the number of studies is small, and the results are preliminary, medium effect sizes have been shown in children and adolescents (García-Escalera et al., 2016).

The Unified Protocol for Transdiagnostic Treatment of Emotional Disorders in Children (UP-C) (Ehrenreich-May et al., 2018) is one of the transdiagnostic CBT treatments for children with emotional disorders. The Unified Protocol (UP) was originally developed for adult patients, and targets emotion dysregulation and negative affectivity, which are believed to be shared risk and maintenance factors for various emotional disorders (Barlow et al., 2017); its efficacy has been thoroughly demonstrated (Sakiris and Berle, 2019; Cassiello-Robbins et al., 2020). The UP-C is a downward extension of the UP, for children. An open trial (Bilek and Ehrenreich-May, 2012) and a randomized controlled trial (RCT) (Kennedy et al., 2019) have examined the feasibility and efficacy of the UP-C, with promising results regarding improvement in anxiety and depressive symptoms. Although there are other transdiagnostic CBT protocols for emotional disorders in children (e.g., Chu et al., 2009, 2016; Weersing et al., 2012; Essau et al., 2014; Martinsen et al., 2016), they are less established than the UP-C and/or are directed more toward preventive goals (García-Escalera et al., 2016).

In Japan, although some school-based prevention programs exist for anxiety and depression (Sato et al., 2009; Ishikawa et al., 2010, 2019; Urao et al., 2018), the only interventions for patients with diagnostic levels of these disorders were CBT program for anxiety disorders (Ishikawa et al., 2012) and avoidance behavior-focused transdiagnostic CBT for anxiety and depressive disorders (Kishida and Ishikawa, 2019), both of which have only shown preliminary efficacy. In addition, the treatment manuals or protocols of these programs are not available to the public, making replication studies difficult. Thus, even though CBT has been shown to be effective in treating emotional disorders in children internationally, there is neither enough data to support this, nor any widely available evidence-based treatment manuals in Japan. Therefore, we considered that introducing the UP-C, which has a treatment manual and is widely applicable to emotional disorders in children, and examining its efficacy would contribute to the dissemination of evidence-based CBT in Japan. In addition, we considered it useful to adapt the UP-C to the Japanese cultural context, since research has indicated the importance of achieving a balance between the selection of scientifically rigorous interventions and a culturally competent practice (Bernal et al., 2009). In fact, a systematic review of UP applications with adult populations showed that the UP has been tested in 11 countries, with numerous adaptations, and these adaptations typically achieved their intended results (Cassiello-Robbins et al., 2020).

This study aimed to develop a Japanese version of the UP-C and examine its feasibility and preliminary efficacy for children (aged 8–12 years) with emotional disorders. Feasibility was evaluated in aspects of safety, by testing the hypothesis that no severe adverse events would occur, and acceptability, by testing the hypotheses that a low dropout and high attendance proportion would be observed and participants would report a sufficient level of program satisfaction. Preliminary efficacy was evaluated by testing the hypothesis that participants would show improvement in the primary outcome at post-treatment (16th week), compared with the pre-treatment, with a large effect size. The primary outcome was the overall severity of emotional disorders as assessed by psychiatrists using the Clinical Global Impression-Severity Scale (CGI-S) (Guy, 1976). Additionally, we hypothesized that anxiety/depressive symptoms and functional status on child- and parent-report questionnaires would improve at post-treatment or follow-up, compared to pre-treatment, based on the prior UP studies for children, adolescents, and adults (Bilek and Ehrenreich-May, 2012; Ehrenreich-May et al., 2017; Kennedy et al., 2019; Sakiris and Berle, 2019).

## Materials and Methods

### Study Design and Procedure

This study used a single-arm pretest, posttest, follow-up design. All procedures were performed in compliance with the Japanese Ethical Guidelines for Medical and Health Research Involving Human Subjects, in addition to the Declaration of Helsinki. The current study’s ethical and scientific validity were approved by the following IRBs: the National Center of Neurology and Psychiatry (approval number: A2016-043) and the National Center for Global Health and Medicine (approval number: NCGM-G-002148-00). This study was registered at the clinical trial registry (UMIN CTR: UMIN000026911).

### Participants

Participants were Japanese children with emotional disorders and their parents, who were seeing child and adolescent psychiatrists in the department of child and adolescent psychiatry at a general hospital in a metropolitan area in Japan. They were recruited through referrals from their psychiatrists between April 2017 and March 2018. The intervention schedule was planned in advance, and participants were recruited. Once the intervention for one group was completed, participants for the next group were recruited accordingly. This procedure was repeated until the required number of participants were registered. Although the UP-C is a program for children aged 6–12 years, this study targeted children in the third to sixth grades (aged 8–12 years), because the speed of writing and understanding among children in lower grades is likely to be different from that of children in higher grades; it would be difficult to combine younger and older children in a group setting. All participants provided written informed consent and assent to participate in this study.

#### Inclusion/Exclusion Criteria

The inclusion criteria were: (a) children with a principal diagnosis of major depressive disorder, persistent depressive disorder, unspecified depressive disorder, separation anxiety disorder, specific phobia, social anxiety disorder, panic disorder, agoraphobia, generalized anxiety disorder, unspecified anxiety disorder, or obsessive-compulsive disorder as per DSM-5^1^; (b) children with CGI-S ≥ 4 at pre-treatment; (c) children in the third to sixth grade at pre-treatment; and (d) children and their parents who gave full consent for participation. The exclusion criteria included: (a) children with a DSM-5 diagnosis of manic or hypomanic episode or psychotic disorders at pre-treatment; (b) children with serious suicidal ideation at pre-treatment; (c) children receiving other structured psychotherapy at pre-treatment or planning to receive it during the intervention; (d) children or parents with severe intellectual disabilities or learning disorders that would interfere with understanding the questions or treatment material; (e) children or their parents who were expected to be absent from at least 5 of 15 sessions; (f) parents with physical, mental, or cognitive disorders that would make it difficult for them to support the child; (g) children with problematic behaviors that might interfere with the implementation of group therapy; and (h) other reason(s) deemed relevant by the investigators. The child and adolescent psychiatrists in charge of each child confirmed the inclusion criteria (a) and (b), and exclusion criteria (a) and (b) based on DSM-5 at pre-treatment^2^. Other criteria were confirmed at the time of obtaining informed consent.

#### Sample Size

In the open trial of the UP-C conducted in the United States (Bilek and Ehrenreich-May, 2012), the pre- to post-treatment effect sizes for principal anxiety disorder severity was Cohen’s *d* = 1.38 and that for the sum of all anxiety and depressive disorder severity ratings was Cohen’s *d* = 1.07. As this is the first study to implement the UP-C in Japan, we conservatively estimated the effect size (Cohen’s *d* = 0.80), referring to previous studies on diagnosis-specific and transdiagnostic CBT for anxiety and depressive disorders in children overseas and in Japan, and calculated the required sample size. When we set the effect size as 0.80, the significance level as 0.05, and the power as 0.80, the sample size required to detect mean differences between the paired two groups was *n* = 15. As the dropout rate for the UP-C in the United States was 18%, the target sample size was set to *n* = 18, by adding the number of people corresponding to that proportion (*n* = 3).

#### Participant Flow and Characteristics

**FIGURE 1:**
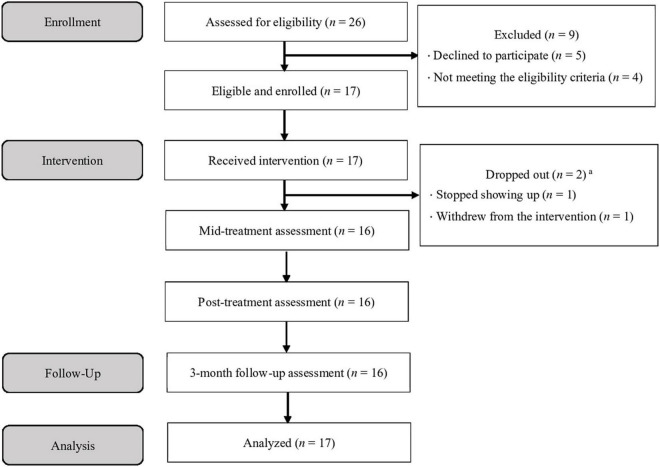
Participant flow diagram. ^a^One patient withdrew from the intervention but completed all assessments.

The participant flow diagram is presented in Figure 1. The child and adolescent psychiatrists referred 26 patients, who were given a description of the study, and ultimately, 17 children^3^ (female *n* = 11, male *n* = 6; *M* = 10.06 ± 0.97 years) and their parents were found to be eligible and agreed to participate in the study (three groups were formed, with eight, five, and four pairs of children and parents, respectively). Among the children, 2 were outpatients and 15 were inpatients. Outpatients were receiving brief supportive psychotherapy sessions and medication as needed from their psychiatrists, while inpatients were receiving these treatments as well as assistance in returning to their home and school in cooperation with the hospital school. These children had been receiving treatment at the hospital for an average of approximately 1 year and 2 months (*M* = 433, *SD* = 377, range = 32–1,358 days) at the time they were enrolled in the study. Seven children (41.2%) were taking psychotropic medication. The parents who participated in the program were mostly mothers (*n* = 16); one father attended alone, and one father attended with the mother.

Table 1 shows the principal and comorbid diagnoses at pre-treatment. The most common primary diagnosis was separation anxiety disorder (*n* = 6, 35.3%). Seven children (41.2%) had at least one comorbid diagnosis (range of comorbid diagnoses = 0–2). Although depressive disorders were also a study target, none of the participants were diagnosed with these disorders.

**TABLE 1:** Principal and comorbid diagnoses at pre-treatment.

	Principal diagnosis *n* (%)	Comorbid diagnoses *n* (%)
Separation anxiety disorder	6 (35.29)	2 (11.76)
Social anxiety disorder	3 (17.65)	1 (5.88)
Panic disorder	–	1 (5.88)
Agoraphobia	–	1 (5.88)
Generalized anxiety disorder	3 (17.65)	–
Unspecified anxiety disorder	2 (11.76)	1 (5.88)
Obsessive-compulsive disorder	3 (17.65)	–
Selective mutism	–	1 (5.88)
Autism spectrum disorder	–	1 (5.88)
Anorexia nervosa	–	1 (5.88)

### Intervention

The intervention was conducted in the hospital. The UP-C is a group CBT program involving 15 group sessions of 90 min each, and the children’s and parents’ sessions are conducted parallelly. The UP-C enables an individual to become an “emotion detective” and solve the mystery of one’s own emotions while enjoying the process. The UP-C encourages children and parents to learn and use the following five skills (referred to as the CLUES skills): C skill = consider how I feel; L skill = look at my thoughts; U skill = use detective thinking and problem solving; E skill = experience my emotions; S skill = stay healthy and happy. Specifically, first, participants learn the skill for increasing awareness of their emotional experiences. Next, they learn about thinking traps and practice identifying the thinking traps they are falling into. The third skill is detective thinking, and they practice using it to get out of their thinking traps. Additionally, they learn problem solving skills to get out of situations where they feel stuck. Fourth, they work on situational emotion exposure individually; this is the most important skill in this treatment. Finally, reviewing the skills learnt thus far, they make a post-treatment plan to prevent relapses. In addition to these five skills, parents learn to monitor both their child’s emotional experiences and their reactions in response to those experiences. They also learn about four emotional parenting behaviors (criticism, overcontrol/overprotection, excessive modeling of strong emotions and avoidance, and inconsistency) that typically exacerbate or maintain emotional disorder symptoms in children, and learn to replace them with opposite parenting behaviors (expressing empathy, healthy independence-granting, healthy emotional modeling, and consistent use of reinforcement and discipline) that are considered effective in managing emotional disorders.

**FIGURE 2:**
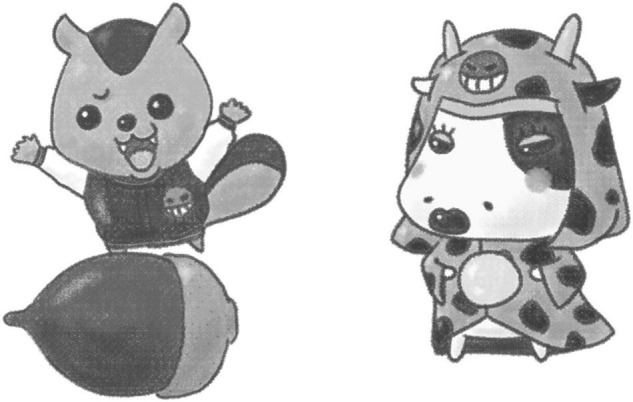
Example of thinking monsters. The monsters represent “jumping to conclusions” (left) and “mind reading” (right). The illustrations were adapted from Ehrenreich-May et al. (2020a).

We used a culturally and linguistically adapted Japanese version of the UP-C. First, we translated the therapist guide and workbook of the UP-C into Japanese. Then, with the developer’s permission, we modified them to increase the acceptability and boost understanding of the treatment, retaining the concept and fundamental contents of the program in the same form as the original version. There were two major modifications in the Japanese version of the UP-C. First, we changed the name of the program and names of the skills. The new program name was chosen to avoid using the words “disorders” and “treatment,” because the stigma attached to mental disorders is still strong in Japan (Ando et al., 2013). Thus, instead of using a direct Japanese translation of the program name (i.e., “Unified Protocol for Transdiagnostic Treatment of Emotional Disorders in Children”), we named the Japanese version the “Emotion Detectives Program for Children.” Regarding the names of the skills, the five emotion management skills are collectively called “CLUES skills” in the original version and are taught one by one as “C skill,” “L skill,” and so on. However, because Japanese children are not familiar with English, such naming does not help them understand or remember these skills. Therefore, for the Japanese version, these five skills were collectively referred to as “emotional detective skills,” and each skill was given detective-themed names, such as “crime scene investigation skill” or “culprit identification skill” (Table 2). Second, we made a modification to the way thoughts are externalized. In the original version, detectives who tend to fall into each thinking trap (i.e., cognitive distortion) appear as thinking trap characters. In the Japanese version, we created unique characters, referred to as “thinking monsters,” to represent each thinking trap. The purpose was to help children learn in an enjoyable way about the thinking traps, which are also difficult for adults to understand, using a character popular among Japanese children, that is, a monster. Figure 2 shows the examples of thinking monsters. Further, the illustrations were adapted to the Japanese culture, and the worksheets were modified to make them easier to understand. There were no major adaptations made to the content for parents. Details of the adaptations are presented elsewhere in the literature (Fujisato et al., 2021), and the Japanese version of the therapist guide and workbook are also available (Ehrenreich-May et al., 2020a,b).

**TABLE 2:** Five Skills of the UP-C: Contents and names in the original and Japanese versions.

Session	Contents	Original skill names	Japanese skill names
1-4	Three aspects of the emotional experience (feelings, thoughts, and behaviors)	C skill: Consider how I feel	Crime scene investigation skill
5	Thinking traps	L skill: Look at my thoughts	Culprit identification skill
6-7	Using detective thinking to get out of thinking traps and working on problem solving	U skill: Use detective thinking and problem solving	Evidence collection and strategy planning skill
8-14	Situational emotion exposures	E skill: Experience my emotions	Confronting skill
15	Relapse prevention	S skill: Stay healthy and happy	Master detective skill

*UP-C, Unified Protocol for Transdiagnostic Treatment of Emotional Disorders in Children.*

### Therapists

All group sessions were conducted by one therapist (a clinical psychologist) dedicated to the children’s group and one therapist (a clinical psychologist) dedicated to the parents’ group. They had 7–10 years of clinical experience, respectively, had conducted UP for adults in about 30–40 cases, and had attended a 2-day training seminar by one of the UP-C developers. Along with these main therapists, one or two co-therapists in the children’s group and one co-therapist in the parents’ group supported the delivery of the sessions.

All sessions were video recorded. Co-therapists rated the adherence and competence of the main therapists for all sessions by using the Adherence/Competency Rating Scale for the UP-C, which was developed by an UP-C developer and modified for the Japanese UP-C version. Adherence items in this scale contain the interventions that should be conducted in children’s and parents’ groups in each session and are rated according to whether the intervention has been implemented (Yes or No). Competence items include the following questions: (a) To what extent was the material from this section delivered flexibly by the clinician(s)?; (b) To what extent was the material from this section delivered with confidence?; and (c) To what extent did the clinician(s) appear to be competent in their delivery of the material? These questions are rated on a Likert-type scale ranging from 0 (delivered inflexibly/no confidence/no competence) to 4 (highly flexible/highly confident/highly competent). Therapist adherence was high (children’s group therapist: 100%; parents’ group therapist: 99.5%). Therapist competence was also high (children’s group therapist: (a) *M* = 3.89, *SD* = 0.32, range = 3–4, (b) *M* = 3.82, *SD* = 0.49, range = 2–4, and (c) *M* = 3.91, *SD* = 0.29, range = 3–4; parents’ group therapist: (a) *M* = 3.89, *SD* = 0.32, range = 3–4; (b) *M* = 3.87, *SD* = 0.34, range = 3–4, and (c) *M* = 3.87, *SD* = 0.34, range = 3–4).

### Measures

The primary outcome measure was the overall rating of the severity of emotional disorders assessed by the psychiatrists in charge of each child using the CGI-S. Secondary outcome measures included the following: improvement of the overall rating of severity of emotional disorders assessed by the psychiatrists using the Clinical Global Impression-Improvement scale (CGI-I) (Guy, 1976); severity of anxiety rated by the children and parents using the Spence Children’s Anxiety Scale (SCAS) (Spence, 1998); severity of depression rated by the children and parents using the Depression Self-Rating Scale for Children (DSRS-C) (Birleson, 1981); and functional status reported by the children and parents using the Child Outcome Rating Scale (CORS) (Duncan et al., 2006). Children’s and parents’ treatment satisfaction were assessed using the Client Satisfaction Questionnaire-8 (CSQ-8) (Larsen et al., 1979).

The assessments by the psychiatrists and the child- and parent-report paper-and-pencil questionnaires were conducted at pre-treatment (-1-week; 1 week before treatment), post-treatment (16-week), and 3 months after the treatment (27-week), considering the first session as 1-week. Further, a mid-treatment assessment (8-week) of child- and parent-report questionnaires alone was conducted (a margin of 2 weeks was allowed). The assessments by the psychiatrists were based on the information provided during consultation in the assessment period. The questionnaires were administered as follows. For the pre-treatment assessment, a different psychologist than the therapists in charge of the sessions was assigned to the participants and helped them complete the questionnaires at the hospital. For the mid- and post-treatment assessment, participants were given the questionnaires during the session and were asked to answer them at home and bring them to the next session. For the 3-month follow-up assessment, the participants visited the hospital and completed the questionnaires.

#### Clinical Global Impression

The CGI is a clinician-rated assessment tool used to determine the severity of illness and improvement following treatment (Guy, 1976). In this study, we used the following two measures, which were translated based on Guy (1976) and Busner and Targum (2007) and modified to fit this study. The CGI-S is a one-item measure for assessing the overall severity of patients’ mental illness. This measure was used to evaluate the overall severity of emotional disorders (i.e., the severity of depressive, anxiety, and obsessive-compulsive disorders) in the children. Severity was rated on a 7-point scale from 1 (= normal, not at all ill) to 7 (= among the most extremely ill patients) based on the rating guidelines, with a higher score indicating a more severe condition. The CGI-I is a one-item measure for assessing overall improvement in patients’ mental illness. This measure was used to evaluate the degree of overall improvement in depressive, anxiety, and obsessive-compulsive symptoms in the children. The degree of improvement was rated on a 7-point scale from 1 (= very much improved) to 7 (= very much worse) based on the rating guidelines; a lower score indicates greater improvement. The evaluator scores the items by considering all of the information obtained at the time of evaluation. The period to be assessed can be set arbitrarily depending on the study using this measure; in the current study, the past week was taken as the assessment period. This scale is an internationally widely used standard measure with good validity (Leon et al., 1993; Berk et al., 2008).

#### Spence Children’s Anxiety Scale

The SCAS is a self-report questionnaire that assesses the severity of anxiety symptoms broadly, in children (Spence, 1998). This scale is based on the DSM-IV-TR and includes items (38 items in total) on separation anxiety (6 items), social phobia (6 items), obsessive-compulsive problems (6 items), panic/agoraphobia (9 items), generalized anxiety/overanxious (6 items), and physical injury fears (specific phobia; 5 items). Respondents were asked to rate the degree to which they experience each symptom on a 4-point frequency scale from 0 (= never) to 3 (= always). A higher total or subscale score indicates more severe anxiety symptoms (total score range = 0-114). The reliability (internal consistency and test-retest reliability) and validity (factorial validity and convergent validity) of the Japanese version of the SCAS have been confirmed (Ishikawa et al., 2009). Furthermore, parents were required to answer the parent version of the SCAS (SCAS-P) (Nauta et al., 2004). The reliability (internal consistency) and validity (factorial validity and convergent validity) of the Japanese version of this scale have been confirmed (Ishikawa et al., 2014). In our sample, internal consistency at pre-treatment was excellent for both child- and parent-reports (Cronbach’s α = 0.94 and 0.95, respectively).

#### Depression Self-Rating Scale for Children

The DSRS-C is an 18-item self-report questionnaire that assesses a child’s depressive symptoms during the past week (Birleson, 1981). Respondents were asked to rate each item on a 3-point scale, from 0 (= never) to 2 (= most of the time). A higher total score indicates more severe depressive symptoms (total score range = 0-36). The reliability (internal consistency and test-retest reliability) and validity (factorial validity and convergent validity) of the Japanese version of the DSRS-C have been confirmed (Murata et al., 1996). In addition to children answering the DSRS-C, parents were also required to assess their child’s depressive symptoms. For parents, we used the same items as the DSRS-C and instructed them to choose options that seemed to be true for their child’s condition during the past week. In our sample, internal consistency at pre-treatment was good for both child- and parent-reports (Cronbach’s α = 0.82 and 0.84, respectively).

#### Child Outcome Rating Scale

The CORS is a self-report questionnaire used to assess a child’s multifaceted functional status (Duncan et al., 2006). This scale consists of four items that inquire about “me” (How am I doing?), family (How are things in my family?), school (How am I doing at school?), and everything (How is everything going?). Each item is rated on a visual analog scale with two icons—one of a frowning face (indicating dysfunction) and the other of a smiling face (indicating good function)—at either end of the line. Respondents were asked to mark where they were located on each 10 cm line segment. Points were scored from 0 to 10, with 0 if the end on the “frowning face” side was marked, and 10 if the end on the “smiling face” side was checked (total score range = 0-40). We contacted Dr. Koji Shiraki, the developer of the Japanese version of this scale, and obtained permission to use the scale (Personal communication, July 15, 2016). Although the Japanese version of the scale has not been validated, this scale is widely used internationally, and the reliability (internal consistency and test-retest reliability) and validity (concurrent validity and construct validity) of the original CORS have been confirmed (Duncan et al., 2006). We also used the CORS for parents and instructed them to indicate where their child was located on each line segment. In our sample, internal consistency for child-reports was low at pre-treatment, but high at post-treatment (Cronbach’s α = 0.34 and 0.82, respectively). Internal consistency for parent-reports was acceptable at both pre- and post-treatment (Cronbach’s α = 0.67 and 0.70, respectively).

#### Client Satisfaction Questionnaire-8

The CSQ-8 is a self-report questionnaire that assesses clients’ satisfaction with the program and consists of eight items (Larsen et al., 1979). Respondents were asked to circle the most applicable of the four response alternatives presented for each item (4-point scale; 1, 2, 3, and 4 points were assigned in ascending order of satisfaction). A higher total score indicated greater satisfaction with the program (total score range = 8-32). The reliability (internal consistency) and validity (criterion validity) of the Japanese version of the CSQ-8 have been confirmed (Tachimori and Ito, 1999). In this study, we used the CSQ-8 to assess parents’ satisfaction with the program, and we used the Client Satisfaction Questionnaire-8-Child and Youth version (CSQ-8-CY) (Tamalpais Matrix Systems, n.d.), an easy-to-understand revised questionnaire for the younger population, to assess children’s satisfaction with the program. We developed the Japanese version of the CSQ-8-CY with the permission of the original developer and implemented it. In our sample, internal consistency was excellent for both child- and parent-reports (Cronbach’s α = 0.96 and 0.93, respectively).

#### Adverse Events

In accordance with the Japanese Ethical Guidelines for Medical and Health Research Involving Human Subjects, adverse events were defined as any undesirable or unintended injuries or illnesses, or signs thereof, occurring in research participants, regardless of whether they were causally related to the research conducted. Of these, those falling under any of the following were judged to be severe adverse events: (1) causing death, (2) life-threatening, (3) requiring hospitalization or extension of the period of hospitalization for treatment, (4) causing permanent or significant disability or malfunction, and (5) causing congenital abnormalities in the offspring. The presence or absence of adverse events was confirmed at each session by inquiring, either verbally or on paper, whether there were any symptoms that had worsened or emerged since the commencement of the program.

### Data Analysis

We performed intention-to-treat (ITT) analyses for all outcome measures. To test the difference between pre-treatment and mid-treatment, post-treatment, and 3-month follow-up results for each outcome, we used a linear mixed model (LMM) with time as a fixed effect and participants as a random effect. Compound symmetry structure was used for the within-subject variance-covariance matrix, and the restricted maximum likelihood method was used to estimate parameters. The missing data were treated as missing with no imputation or exclusion. Thus, all data including missing data were used in the estimation with restricted maximum likelihood estimation. The adjusted means for each time point estimated from the LMM were used to test the difference in mean scores between time points (Bonferroni correction). We also calculated the effect sizes (Hedges’ *g* and its 95% confidence intervals) for the change in outcomes between pre-treatment and mid-treatment, pre-treatment and post-treatment, and pre-treatment and 3-month follow-up, using the adjusted means estimated by the LMM. In addition, to determine whether changes in symptom levels were clinically significant, we calculated the proportion of participants’ treatment response, where treatment response status was defined as a CGI-I score of “1 = very much improved” or “2 = much improved,” as in previous studies (Kennedy et al., 2019). The effect size calculator of langtest.jp was used to calculate the effect sizes and SPSS Statistics version 24 was used for the other analyses. *P* < 0.05 (two-tailed) was considered statistically significant.

## Results

### Feasibility

Fifteen adverse events were recorded during the study. These included irritability, difficulty sleeping, fatigue, and restlessness. There were no severe adverse events. Of the 17 participants, 2 dropped out (11.76%; after the 1st and 4th sessions). Independent sample *t*-tests revealed that all pre-treatment scores for the two dropout participants did not differ significantly from the completers (i.e., those who participated in the intervention till the end without dropping out) (*t* = 0.06–1.23, *p* = 0.24–0.95). The proportion of the completers’ attendance was 95.6% in children (*M* = 14.3, *SD* = 0.87, range = 13–15) and 94.6% in parents (*M* = 14.2, *SD* = 1.11, range = 12–15). In addition, the level of satisfaction for the UP-C assessed using the CSQ-8 was above an average of 3 of 4 points per item for both children and parents (children: *M* = 24.93, *SD* = 7.64, range = 8–32; parents: *M* = 27.00, *SD* = 3.98, range = 19–32). The means and standard deviations for each item of the CSQ-8 are shown in Supplementary Table 1. These findings indicate that the Japanese version of the UP-C was favorably received by the participants; the “thinking monsters” were especially popular among them.

### Treatment Outcomes

Table 3 shows the means and standard deviations of outcome measures at pre-treatment, mid-treatment, post-treatment, and 3-month-follow-up, as well as the results of examining the differences in means, using the LMM. The effect sizes (Hedges’ *g*) and their 95% confidence intervals are presented in Table 4.

**TABLE 3:** Scores of outcomes and the differences in scores between pre-treatment and other time points.

	Mean (*SD*)				Multiple comparisons
	Pre	Mid	Post	FU	
**Clinician-report**					
CGI-S	4.65 (1.05)	–	3.53 (1.05)	3.24 (1.05)	Pre > Post**, FU**
CGI-I	–	–	2.47 (0.91)	2.53 (0.91)	
**Child-report**					
SCAS	46.53 (21.90)	37.08 (22.19)	30.90 (22.19)	22.71 (22.19)	Pre > Mid*, Post**, FU**
DSRS-C	12.77 (5.94)	10.97 (6.08)	11.56 (6.08)	8.78 (6.08)	Pre > FU*
CORS	22.10 (8.43)	24.45 (8.64)	26.02 (8.64)	28.05 (8.64)	Pre < FU*
**Parent-report**					
SCAS	47.35 (21.13)	38.88 (21.44)	34.00 (21.44)	22.00 (21.44)	Pre > Post**, FU**
DSRS-C	12.71 (5.27)	10.85 (5.37)	10.92 (5.37)	14.35 (5.37)	
CORS	20.14 (7.18)	27.44 (7.39)	27.01 (7.39)	27.23 (7.39)	Pre < Mid**, Post**, FU**

*Pre, pre-treatment; Mid, mid-treatment; Post, post-treatment; FU, 3-month follow-up; CGI-S, Clinical Global Impression-Severity Scale; CGI-I, Clinical Global Impression-Improvement Scale; SCAS, Spence Children’s Anxiety Scale; DSRS-C, Depression Self-Rating Scale for Children; CORS, Child Outcome Rating Scale.*

**p < 0.01, *p < 0.05.

**TABLE 4:** Effect sizes of outcomes (Hedges’ *g*, 95% CI).

	Pre to Mid	Pre to Post	Pre to FU
**Clinician-report**			
CGI-S	–	1.04 (0.31 to 1.77)	1.31 (0.56 to 2.07)
**Child-report**			
SCAS	0.42 (−0.27 to 1.11)	0.69 (−0.01 to 1.40)	1.05 (0.32 to 1.79)
DSRS-C	0.29 (−0.39 to 0.98)	0.20 (−0.49 to 0.88)	0.65 (−0.05 to 1.35)
CORS	−0.27 (−0.95 to 0.42)	−0.45 (−1.14 to 0.24)	−0.68 (−1.38 to 0.02)
**Parent-report**			
SCAS	0.39 (−0.30 to 1.08)	0.61 (−0.09 to 1.31)	1.16 (0.42 to 1.90)
DSRS-C	0.34 (−0.35 to1.03)	0.33 (−0.36 to 1.02)	−0.30 (−0.99 to 0.39)
CORS	−0.98 (−1.70 to −0.25)	−0.92 (−1.64 to −0.20)	−0.95 (−1.67 to −0.23)

*Pre, pre-treatment; Mid, mid-treatment; Post, post-treatment; FU, 3-month follow-up; CGI-S, Clinical Global Impression-Severity Scale; SCAS, Spence Children’s Anxiety Scale; DSRS-C, Depression Self-Rating Scale for Children; CORS, Child Outcome Rating Scale.*

#### Primary Outcome

The CGI-S scores significantly improved at post-treatment (*MD* = −1.12, 95% CI = −1.76 to −0.472, *p* = 0.001) and the 3-month follow-up (*MD* = −1.41, 95% CI = −2.06 to −0.77, *p* = 0.000) compared with pre-treatment. Large effect sizes were observed both from pre-treatment to post-treatment (*g* = 1.04, 95% CI = 0.31–1.77) and pre-treatment to the 3-month follow-up (*g* = 1.31, 95% CI = 0.56–2.07).

#### Secondary Outcomes

The SCAS scores significantly improved at post-treatment (child-report: *MD* = −15.63, 95% CI = −25.07 to −6.20, *p* = 0.000; parent-report: *MD* = −13.35, 95% CI = −23.05 to −3.66, *p* = 0.004) and the 3-month follow-up (child-report: *MD* = −23.82, 95% CI = −33.26 to −14.38, *p* = 0.000; parent-report: *MD* = −25.35, 95% CI = −35.05 to −15.66, *p* = 0.000) compared with pre-treatment, in both children’s and parents’ reports with medium to large effect sizes (*g* = 0.61–1.16). Additionally, child-reported CORS scores improved gradually throughout the study period and were significantly higher at the 3-month follow-up compared with pre-treatment (*MD* = 5.95, 95% CI = 0.37–11.53, *p* = 0.033) with a medium effect size (*g* = 0.68). Parent-reported CORS scores significantly improved at mid-treatment compared with pre-treatment (*MD* = 7.30, 95% CI = 2.00–12.61, *p* = 0.004), and this treatment effect was maintained during the post-treatment (*MD* = 6.86, 95% CI = 1.56–12.17, *p* = 0.007) and the 3-month follow-up period (*MD* = 7.09, 95% CI = 1.79–12.39, *p* = 0.005) with large effect sizes (*g* = 0.92–0.98). However, although child-reported DSRS-C scores significantly improved at the 3-month follow-up compared with pre-treatment (*MD* = −3.98, 95% CI = −7.71 to −0.25, *p* = 0.033) with a medium effect size (*g* = 0.65), there were no significant differences between pre-treatment and other time points in the parents’ reports.

#### Treatment Response

Of the 15 participants, 9 achieved treatment response status (60.0%), both at post-treatment and at the 3-month follow-up, when only participants who completed the treatment were included. When examined using ITT sample, of the 17 participants, 10 achieved treatment response status at post-treatment (58.8%) and 9 at the 3-month follow-up (52.9%).

## Discussion

This study aimed to examine the feasibility and preliminary efficacy of the Japanese version of the UP-C. Feasibility was demonstrated in terms of a low proportion of dropouts (2/17 participants, 11.76%), a high proportion of completers’ attendance (children: *M* = 14.3/15 sessions, 95.6%; parents: *M* = 14.2/15 sessions, 94.6%), a sufficient program satisfaction level, and no severe adverse events. The results also showed preliminary efficacy of the Japanese version of the UP-C in improving the overall severity of emotional disorders, severity of anxiety symptoms, and functional status in Japanese children with emotional disorders.

### Feasibility of the Japanese Version of the UP-C

It was hypothesized that the Japanese version of the UP-C would be feasible for Japanese children with emotional disorders and their parents, with a lack of severe adverse events, low dropout proportion, high attendance proportion, and sufficient program satisfaction. Strong support was found for this hypothesis. No severe adverse event was observed during the intervention and follow-up period, indicating the potential safety of the Japanese version of the UP-C. The dropout proportion of 11.76% was lower than that of the open trial of the UP-C conducted in the United States (18.18%) (Bilek and Ehrenreich-May, 2012). Additionally, in this study, completers’ attendance was remarkably high. All participants, except for the two dropouts, attended at least 11 sessions to be defined as a treatment completer in the abovementioned open trial; the 88% attendance rate in the current study exceeded the 74% reported in the United States trial. Both children and parents reported a sufficient degree of satisfaction with the program, as the CSQ-8 item mean score was above the third point of the scale, which is “satisfied.” However, compared to other trials for children and parents (e.g., Weisz et al., 2017; Lebowitz et al., 2020), child-rated satisfaction in this study tended to be somewhat lower, and SD was higher. In a transdiagnostic group therapy setting, therapists must deal with a highly diverse group of children. It is possible that the needs of individual children were not completely met. As the CSQ-8 has not been employed in trials using CBT with children in Japan, we cannot draw any conclusions based on previous studies; however, detailed examinations of children’s satisfaction in future studies are necessary.

In general, these findings suggest that the Japanese version of the UP-C is acceptable for children with emotional disorders and their parents in Japan. It has been pointed out that achieving a balance between the selection of scientifically rigorous interventions and a culturally competent practice is important when introducing treatments developed overseas (Bernal et al., 2009); thus, adapting the UP-C to the Japanese culture appears to have been effective.

### Preliminary Efficacy of the Japanese Version of the UP-C

It was hypothesized that the participants would show improvement in the primary outcome, based on the CGI-S ratings, at post-treatment compared to pre-treatment. This hypothesis was strongly supported. The CGI-S scores decreased significantly from pre- to post-treatment, with a large effect size. This indicates that the Japanese version of the UP-C can improve overall severity of emotional disorders. In addition, this treatment effect was maintained during the 3-month follow-up period.

Additionally, it was predicted that anxiety/depressive symptoms and functional status would improve at post-treatment or follow-up, compared to pre-treatment. Moderate support was found for this hypothesis. Results indicated that child- and parent-reported anxiety symptoms improved over time. At the 3-month follow-up, child-reported anxiety symptoms were the same as the average symptoms of Japanese elementary school students (*M* = 23.5, *SD* = 18.75) (Ishikawa et al., 2009). Whereas, child-reported depressive symptoms improved from pre-treatment to the 3-month follow-up, but there were no significant differences in the parents’ reports between pre-treatment and other assessment points. As none of the participants had depressive disorders, and the mean score of the DSRS-C at pre-treatment was lower than the cut-off point of 16 on the Japanese version of the DSRS-C (Murata et al., 1996), it is likely that there was little change in depressive symptoms that could be observed by parents. However, the scores of parent-reported depressive symptoms seemed to increase at 3-month follow-up compared with post-treatment, which needs to be carefully considered and examined in future studies. The course of change in the functional status of the children seemed to differ between children’s and parents’ reports. Results revealed that parents perceived functional changes in their children relatively early in the intervention, while children themselves perceived these changes after the intervention had been completed. This indicates that even if changes are immediately obvious to others, children may take longer to perceive these changes themselves.

Finally, the proportion of treatment response in this study was lower, especially at follow-up, compared to a RCT of the UP-C conducted in the United States (Kennedy et al., 2019) (post-treatment: 58.8% vs. 62.5%; follow-up: 52.9% vs. 75.0%). As the participants in this study were outpatients and inpatients of a child psychiatry department of a general hospital in a metropolitan area, they may have had more severe symptoms than the participants in the RCT mentioned above, which recruited participants through flyers and list-serve announcements and was conducted in a university setting. Alternatively, treatment response may have been affected by the different follow-up periods (3-month vs. 6-month) or the people conducting symptom assessments (psychiatrists in charge of each child vs. blinded independent evaluators).

### Limitations and Future Directions

The results of this study indicated the feasibility and preliminary efficacy of the Japanese version of the UP-C for children with emotional disorders in Japan. However, as this was a pilot study, several limitations should be considered when interpreting the results. First, the sample size and study design employed in this study were insufficient to reach conclusions about efficacy. As this was a single-arm study without a control group, we cannot rule out the possibility that factors such as time course or other factors besides the intervention may have affected the degree of symptom improvement. In addition, owing to the small sample size, the results of this study need to be interpreted within a range of confidence intervals. For the primary outcome, the effect size was large, and the confidence interval did not include zero, indicating that this result is stable. Second, there are some biases in the sample. This study was conducted on patients in the child psychiatry department of a general hospital in a metropolitan area. As such, it is unclear whether similar results would be obtained in other regions or settings. In the future, we suggest conducting multicenter studies including various regional facilities in different settings. Additionally, patients with primary depressive disorders were targeted in this study; however, in fact, such patients were not included. The patients in the sample were not diagnosed with any depressive disorder. An open trial conducted in the United States (Bilek and Ehrenreich-May, 2012) also did not include participants with a primary depressive diagnosis. Considering the low prevalence of these disorders in this age group, these results are somewhat reasonable. However, six participants (35.3%) reported experiencing elevated depressive symptoms, as indicated by a score equal to or greater than 16 (cut-off point in Japan) on the DSRS-C (Murata et al., 1996). Treatments that can be administered without excluding children with these symptoms would be greatly beneficial. Nonetheless, it is certainly necessary to verify these results including patients with a primary diagnosis of depressive disorders in the future. Further, although the UP-C is a treatment program for children aged 6–12 years, this study targeted children aged 8–12 years. Therefore, it is necessary to examine whether the Japanese version of the UP-C is also feasible and effective for younger children. Finally, while conducting the diagnoses, we did not use a standardized diagnostic interview but instead adopted diagnoses made by psychiatrists, from the perspective of cost-effectiveness. In a meta-analysis (Rettew et al., 2009), it was found that diagnostic agreement between standardized diagnostic interviews and clinical evaluations was low to moderate for most disorders. Considering a comparison with other studies, it may be desirable to use standardized diagnostic interviews for diagnosis in future studies.

Despite these limitations, it is important to note that this was the first study to examine the feasibility and preliminary efficacy of the UP-C for children with emotional disorders outside the United States, where the program was developed. In addition, this study included inpatients and patients with comorbid non-emotional disorders (i.e., autism spectrum disorder and anorexia nervosa). These patients completed treatment, and the results were generally favorable. It is promising that the feasibility and preliminary efficacy of the UP-C were confirmed in this study, which was conducted in a setting relatively close to the actual clinical environment without excluding these patients. If the UP-C proves to be feasible and effective in Japan, the clinical implications could be significant; it could greatly contribute to disseminating evidence-based CBT for children with emotional disorders in Japan. As a program that can simultaneously target various symptoms with just one protocol, the UP-C has potential benefits both for patients and therapists and can help alleviate symptoms in Japanese patients efficiently. A stricter RCT that addresses the limitations of this study should be conducted in future to further evaluate this possibility.

## Data Availability Statement

The datasets presented in this article are not readily available because participants of this study did not agree for their data to be shared publicly. Requests to access the datasets should be directed to corresponding author.

## Ethics Statement

The studies involving human participants were reviewed and approved by the National Center of Neurology and Psychiatry (approval number: A2016-043) and the National Center for Global Health and Medicine (approval number: NCGM-G002148-00). Written informed consent to participate in this study was provided by the participants’ legal guardian/next of kin.

## Author Contributions

HF, NK, MI, MU, and MH designed the study. MU contributed to the recruitment of participants. HN was in charge of the clinical assessment. HF, NK, TN, and SN were in charge of the program sessions. HF conducted the statistical analysis and wrote the first draft of the manuscript. NK, HN, MI, MU, TN, SN, and MH provided critical comments on the manuscript related to intellectual content. All authors read and approved the final manuscript.

## Conflict of Interest

HF, NK, MI, and MH published a treatment workbook and a therapist guide for the Japanese version of the UP-C but did not receive any royalties from Fukumura Shuppan. There is a possibility for these authors to receive royalties if these books exceed 900 and 750 prints, respectively. MI received royalties from Shindan to Chiryo Sha (which includes royalties for treatment manuals and related books for the UP), Sogensha, and several publishing companies in Japan. He also received consultant fees from TIS Inc. MH received consultant fees from Mitsubishi Tanabe Pharma and TIS Inc., as well as research grants from Mitsubishi Tanabe Pharma and Jolly Good Inc. The remaining authors declare that the research was conducted in the absence of any commercial or financial relationships that could be construed as a potential conflict of interest.

## Publisher’s Note

All claims expressed in this article are solely those of the authors and do not necessarily represent those of their affiliated organizations, or those of the publisher, the editors and the reviewers. Any product that may be evaluated in this article, or claim that may be made by its manufacturer, is not guaranteed or endorsed by the publisher.

## Footnotes

1 The UP-C study conducted in the United States (Bilek and Ehrenreich-May, 2012; Kennedy et al., 2019) included posttraumatic stress disorder (PTSD) as one of the diagnoses that is eligible for inclusion (although none of the actual participants had PTSD); however, we did not include PTSD because we were advised by the UP-C developer that it is somewhat difficult to treat patients with PTSD together with patients with other emotional disorders in a group and because PTSD was treated with trauma-focused CBT at the hospital where the study was conducted.

2 The psychiatrists were asked to make the diagnosis based on the DSM-5 criteria but were not asked to use a structured interview.

3 As described in section “Sample Size,” the number 18 was based on the expectation that 3 participants would drop out, and the actual number needed was 15. In this study, only 2 participants dropped out, and the study was completed with 17 people because the required number of participants completed the intervention.
