# Supplementary material for: The Unified Protocol for Transdiagnostic Treatment of Emotional Disorders Among Japanese Children: A Pilot Study
Source: Front Psychol. 2021 Nov 25;12:731819. doi: 10.3389/fpsyg.2021.731819 (PMC8654783; doi:10.3389/fpsyg.2021.731819)
Supplement: Supplementary file 1 [file Table_1.pdf]

Supplementary Table 1. Descriptive Statistics for Satisfaction with the Program

Items (response alternatives)		*Mean* (*SD*)	
		Child-report	Parent-report
1	How would you rate the quality of service you have received?<br>(1: Poor, 2: Fair, 3: Good, 4: Excellent)	3.13 (1.25)	3.67 (0.49)
2	Did you get the kind of service you wanted?<br>(1: No, definitely not, 2: No, not really, 3: Yes, generally, 4: Yes, definitely)	2.87 (1.13)	3.13 (0.64)
3	To what extent has our program met your needs?<br>(1: None of my needs have been met, 2: Only a few of my needs have been met, 3: Most of my needs have been met, 4: Almost all of my needs have been met)	3.27 (1.10)	2.80 (0.86)
4	If a friend were in need of similar help, would you recommend our program to him or her?<br>(1: No, definitely not, 2: No, I don't think so, 3: Yes, I think so, 4: Yes, definitely)	3.13 (1.06)	3.60 (0.51)
5	How satisfied are you with the amount of help you received?<br>(1: Quite dissatisfied, 2: Indifferent or mildly dissatisfied, 3: Mostly satisfied, 4: Very satisfied)	3.40 (0.91)	3.20 (0.56)
6	Have the services you received helped you to deal more effectively with your problems?<br>(1: No, they seemed to make things worse, 2: No, they really didn't help, 3: Yes, they helped, 4: Yes, they helped a great deal)	3.07 (0.80)	3.40 (0.63)
7	In an overall, general sense, how satisfied are you with the service you have received?<br>(1: Quite dissatisfied, 2: Indifferent or mildly dissatisfied, 3: Mostly satisfied, 4: Very satisfied)	3.20 (1.08)	3.60 (0.63)
8	If you were to seek help again, would you come back to our program?<br>(1: No, definitely not, 2: No, I don't think so, 3: Yes, I think so, 4: Yes, definitely)	2.87 (1.25)	3.60 (0.51)
